# Disseminated Cerebrospinal Embryonal Tumor in the Adult

**DOI:** 10.1155/2016/6785459

**Published:** 2016-10-13

**Authors:** Alessandro Caporlingua, Daniele Armocida, Federico Caporlingua, Gennaro Lapadula, Grazia Maria Elefante, Manila Antonelli, Maurizio Salvati

**Affiliations:** ^1^Department of Neurology and Psychiatry, Neurosurgery, Sapienza University of Rome, Rome, Italy; ^2^Department of Radiological, Oncological and Anatomo-Pathological Sciences, Sapienza University of Rome, Rome, Italy; ^3^Department of Science and Medical Surgical Biotechnology, Sapienza University of Rome, Rome, Italy

## Abstract

*Introduction*. According to the 2016 World Health Organization classification of Tumors of the Central Nervous System, the term Primitive Neuroectodermal Tumor has been replaced by the term Embryonal Tumor (ET). We present a case of disseminated cerebrospinal ET presenting in an adult patient.* Illustrative Case*. A 49-year-old male presenting with low back pain, dysuria, and hypoesthesia of the lower extremities referred to our emergency department. Brain and whole spine contrast-enhanced MRI documented a diffusively disseminated heterogeneous neoplasm with intradural extra- and intramedullary involvement of the cervicothoracic tract and cauda equina. A primary biopsy of the lumbosacral localization was performed through L5 bilateral laminectomy. Histologic diagnosis was Embryonal Tumor Not Otherwise Specified. The patient underwent chemotherapy with postoperative adjuvant alternating Vincristine-Doxorubicin-Ifosfamide (VAI) and Ifosfamide-Etoposide (IE).* Discussion*. Spinal ETs are exceedingly rare especially when presenting in the adult patient. Neurosurgical and oncologic management is still unclear. When feasible, surgical removal should always be performed to obtain a histologic diagnosis. Postoperative adjuvant therapy might entail both chemo- and radiotherapy; however a consensus on this matter is still lacking.

## 1. Introduction

According to the 2016 World Health Organization (WHO) Tumors of the Central Nervous System (CNS) classification, the term “Primitive Neuroectodermal Tumors (PNETs)” has been removed from the diagnostic lexicon. A newly defined category of CNS tumors, including PNETs among other nosological entities, according to the old nomenclature, has been introduced under the term Embryonal Tumors (ETs) [1]. The presence of amplification of the 19q13.42 region on chromosome 19 (C19MC) differentiates between Embryonal Tumors with Multilayered Rosettes (ETMR), C19MC altered, and ETMR, C19MC nonaltered, or Not Otherwise Specified (NOS). ETs are malignant small, highly cellular neoplasms occurring predominantly in children, rarely in adults. The most common localization of the disease is the cerebellum (medulloblastomas) accounting for 20–25% of all pediatric brain tumors [2]. ETs primarily occur in children and young adults, usually before the fourth decade, with a mean age of 20 years. ETs are described as arising also in the pineal region, cortex, brain stem, and peripheral nerves in adults. A primary intraspinal ET is rare; the mainstay of therapy is a surgical resection whenever feasible followed by chemo- and radiotherapy; a consensus regarding the most adequate adjuvant treatment protocol is not available up to the present date [3].

Intraspinal ETs are rapid-growing soft tissue masses, which cause symptoms of nerve compression, ranging from radiculopathy to sphincter dysfunction, and pain. They are highly malignant and invasive, with a high rate of relapse, and retain a poor prognosis. The five-year survival rate is 30–40% and the median survival ranges from 12 to 24 months; prognosis of these tumors in adults is somewhat worse when diagnosed in adults [3–5].

We report one case of a diffuse ET with brain and spinal dissemination in a 49-year-old male presenting with low back pain, numbness of the lower extremities, and sphincter dysfunction which represented not only a diagnostic dilemma given its aspecific clinical presentation and peculiar radiological features but also, and most importantly, a therapeutic challenge given the lack of consensus regarding its management.

## 2. Illustrative Case

A 49-year-old man presented to our emergency department in October 2015, describing numbness of the lower extremities and low back pain developed during the previous two weeks. Furthermore, he complained of dysuria during the last few days and progressive subjective wasting of lower limbs muscles over the last months. Patient family history and general physical examination did not provide any contributive information. Neurologic examination confirmed a diffuse hypotrophy of lower limbs muscles, without any focal motor deficit and associated with hypoesthesia of the sciatic territory bilaterally.

**Figure 1:**
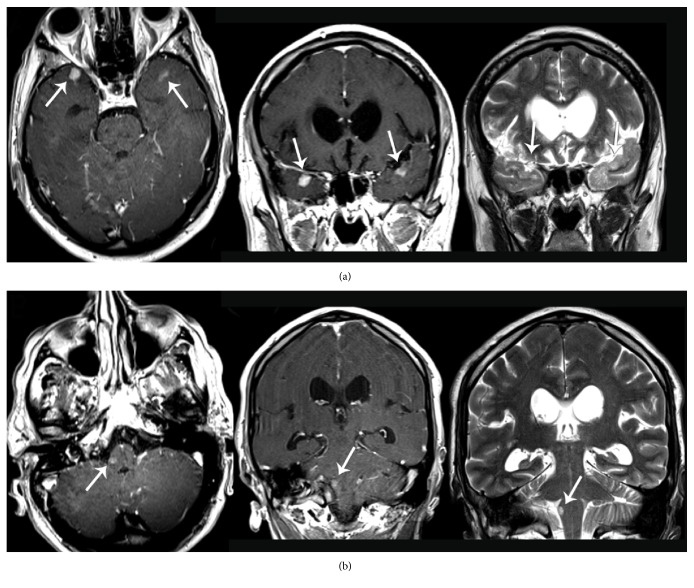
Contrast-enhanced T1- and T2-weighted brain MRI sequences showing contrast enhancing infracentimetric nodules of both temporopolar regions (line (a)) and of the brainstem (line (b)) (white arrow).

**Figure 2:**
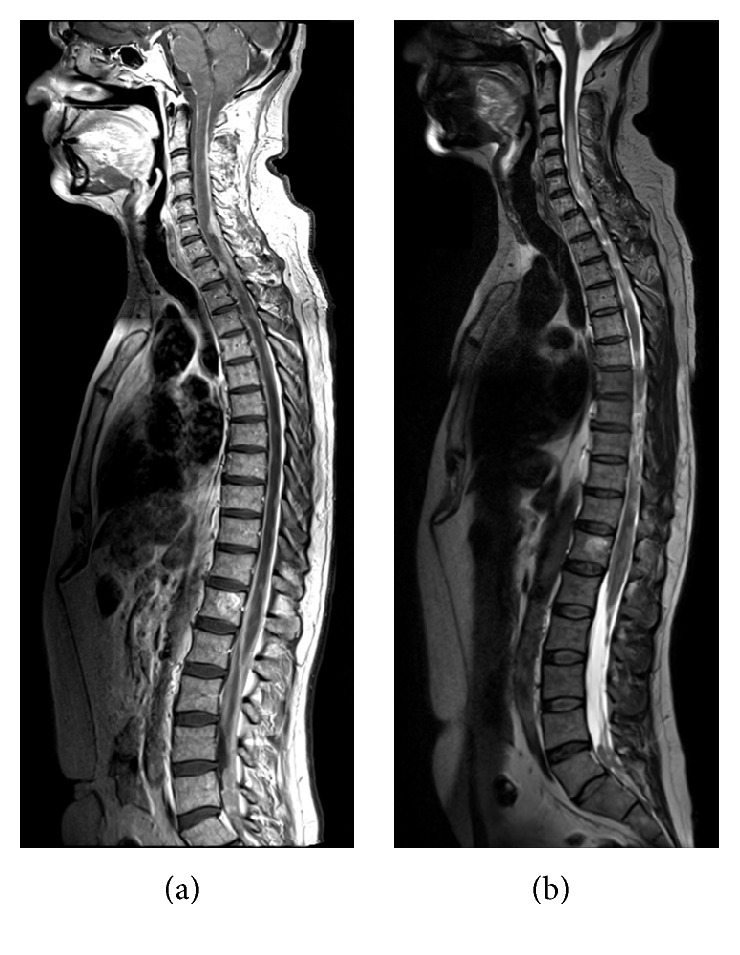
Contrast-enhanced T1- (a) and T2-weighted (b) whole spine MRI sequences showing disseminated intradural intra- and extramedullary Embryonal Tumor of the cervicothoracic tract and cauda equina.

A brain MRI (Figure 1) showed multiple infracentimetric corticosubcortical nodular lesions of temporopolar regions bilaterally of the medulla oblongata, cerebellar tonsils, and left cerebellar hemisphere which appeared hyperintense on T2-weighted sequences and presented a homogenous enhancement on T1 contrast-enhanced sequences. A whole spine MRI (Figure 2) documented a diffuse leptomeningeal contrastografic enhancement with numerous irregularly shaped intra and extramedullary lesions of the midcervical to cervicothoracic junction and lumbosacral tracts, corresponding to a diffuse medullary hyperintensity on T2-weighted sequences. The roots of the cauda equina were enlarged with signal alteration on both T2-weighted and contrast-enhanced sequences suggesting diffuse neoplastic infiltration. A spectroscopic analysis performed on the left cerebellar subcortical lesion showed peaks of lipid/lactate, reduction of N-acetyl-aspartate (NAA), and increase in choline (CHO) suggesting a discariocinetic injury.

A cerebrospinal fluid (CSF) sample was collected; however no neoplastic cells were documented on cytological examination.

The patient underwent a primary biopsy on the lumbosacral localization which appeared to enlarge the S1-S2 posterior sacral foramen causing compression of the right S2 nerve root. Using a posterior approach, a central L5 laminectomy along with median durotomy was performed exposing a grey, soft, hemorrhagic mass which completely enveloped the roots of the cauda equina.

Tissues samples were collected for histological examination.

Patient was discharged on the ninth postoperative day and referred to our oncologic unit. Chemotherapy with alternating Vincristine-Doxorubicin-Ifosfamide (VAI) and Ifosfamide-Etoposide (IE) was the adjuvant treatment protocol of choice which the patient is currently undertaking with a satisfying profile of tolerability.

## 3. Histological Examination

**Figure 3:**
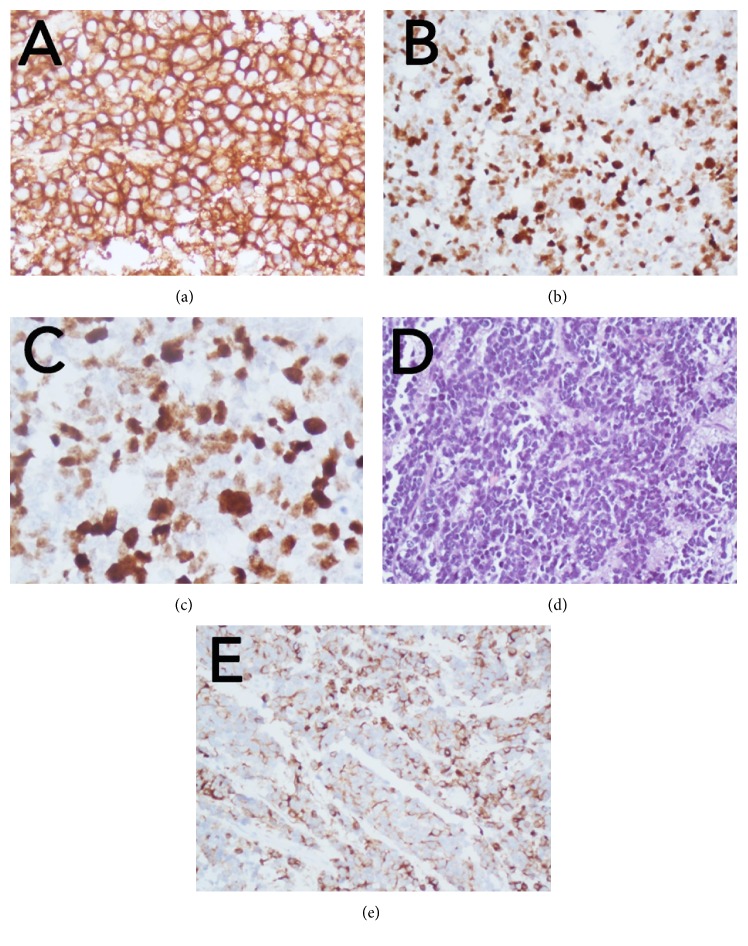
(a) Neoplastic cells show diffuse and strong immunoreactivity for synaptophysin; (b-c) high proliferation activity is highlighted by many Ki-67 immunoreactive tumor cell nuclei. (d) H&E image of a highly cellular neoplasm, composed of small- to medium-sized cells with hyperchromatic nuclei and little cytoplasm; rosette formation is rarely seen; mitotic figures and necrosis of individual tumor cells are typical features. (e) Focal glial fibrillary acid protein (GFAP) immunoreactivity in individual cells; many tumor cells are negative.

Intraoperative smear cytopathological examination showed a “small blue cell” neoplasm, composed of sheets of discohesive undifferentiated, hyperchromatic cells, with occasional evidence of Homer Wright rosette formation. Histological examination on formalin-fixed, paraffin-embedded sections revealed a highly cellular neoplasm composed of small- to medium-sized cells with little perinuclear cytoplasm and hyperchromatic nuclei with granular chromatin. Mitoses were frequent, as was necrosis of single tumor cells. Immunohistochemistry showed an intense and diffuse positivity for Neural Cell Adhesion Molecule (N-CAM) and a slightly less intense, but almost equally diffuse positivity for glial fibrillary acid protein (GFAP). Expression of protein INI1 was detected while LIN28 was not detected; moreover no amplification of 19q13.42 was detected. These features allowed excluding both an Atypical Teratoid Rhabdoid Tumor (AT/RT) and Embryonal Tumor with Multilayered Rosettes (ETMR), respectively. Proliferation index was high (Ki-67 over 40%). Reactions with antibodies against synaptophysin, keratin, and epithelial membrane antigen (EMA) were negative in all tumor cells (Figure 3).

According to the old WHO classification of Tumors of CNS, this tumor could be categorized as a PNET of central origin; however, since the introduction of the 2016 classification, this lesion could not be defined neither as a PNET (the latter being completely removed from current classifications) nor as the newly defined Embryonal Tumors with Multilayered Rosettes (ETMR), leaving the Embryonal Tumor Not Otherwise Specified (NOS) as the sole diagnostic option.

## 4. Discussion

The term Primitive Neuroectodermal Tumor (PNET) was created in 1973 by Hart and Earle to describe predominantly undifferentiated tumors of the cerebrum which contained 90–95% of undifferentiated cells not meeting diagnostic criteria for other tumor entities [6]. First case of PNET dates back to 1969 [7]. Ten years later, Rorke, Becker, and Hinton independently advocated that all central nervous system tumors predominantly composed of primitive neuroepithelial cells should be called PNETs [8, 9]. According to the World Health Organization classification of Tumors of the Central Nervous System, renewed in 2016, the term PNETs no longer exists. All PNETs are to be categorized under the term Embryonal Tumor (ET) including Embryonal Tumors with Multilayered Rosettes (ETMR), Atypical Teratoid Rhabdoid Tumor (AT/RT), and Not Otherwise Specified (NOS) [1].

Primary spinal ETs are more frequently observed in infants, children, and young adults (age range 1 to 70 years; mean 22.5 years). The duration of symptoms, initially entailing radiculopathy and paresthesia, may range from one week to six months. Most cases of primary spinal ETs, according to the old classification, retain a peripheral origin (expression of CD99) and rarely present a central origin (CD99 not expressed). Despite being extremely useful for differentiating between ETs of central or peripheral origin, the expression of CD99 should always be associated with the research of (11; 22) (q24; q12) translocation, which is diagnostic for ETs of peripheral origin typically related to Ewing sarcoma [3].

Imaging features are nonspecific, including intradural intra- and/or extramedullary more or less diffuse lesions showing heterogeneous patterns of contrast enhancement (including diffuse leptomeningeal enhancement), central necrosis, and hemorrhage. The mainstay of treatment is surgical resection; however, this is uncommonly feasible due to the multifocal extensive dissemination of the disease which is not uncommon at presentation. The adjuvant treatment protocol should include a combination of chemo- and radiotherapy; however a consensus on this subject is still lacking due to the exceeding rarity of the disease preventing drawing meaningful conclusions on the basis of the currently available short case-series or single case reports in the literature.

Most cases of ETs involving the spinal cord are “drop” metastases from primary intracranial tumors. Our patient showed an extremely diffuse spinal disease with few intracranial nodules (Figures 1 and 2), a figure normally associated with a worst prognosis. In most patients cited in the literature, spinal localization involved the cauda equina with a single report of supratentorial dissemination. ETs of central origin such as our case are rare proliferative and highly aggressive neoplasms. Their presentation in the adult with major involvement of the intradural extramedullary compartment is extremely rare. Spinal cord compression or cauda equina causes the symptoms that lead to diagnosis. To date, a spinal ET with diffuse cervicothoracic-lumbosacral and intracranial dissemination was never reported.

According to our experience, an open-field biopsy of the lumbosacral spine localization was the least risky method to obtain a histological diagnosis in this specific case considering both the reduced size of brain multifocal nodules and the unforeseeable risk of hemorrhagic complications of any needle biopsy procedure.

Case reporting, for such rare nosological entity, may be extremely useful not only for further understanding of Embryonal Tumors but also for providing evidences for future reviews aiming to define a consensus regarding the therapeutic management of this malignant disease.
